# Assessment of iron metabolism and iron deficiency in incident patients on incident continuous ambulatory peritoneal dialysis

**DOI:** 10.1515/med-2024-1035

**Published:** 2024-09-17

**Authors:** Qinghua Yin, Na Guo, Ping Fu, Hui Zhong

**Affiliations:** Kidney Research Institute, Division of Nephrology, West China Hospital, Sichuan University, Chengdu 610041, Sichuan, China; Division of Nephrology, Department of Pediatrics, West China Second Hospital, Sichuan University, Chengdu 610041, Sichuan, China; Kidney Research Institute, Division of Nephrology, West China Hospital, Sichuan University, No. 37 Guoxue Alley, Chengdu 610041, Sichuan, China

**Keywords:** peritoneal dialysis, serum ferritin, ferritin saturation, iron deficiency, inflammation

## Abstract

**Objective:**

The aim of this study was to investigate iron status and iron deficiency in incident continuous ambulatory peritoneal dialysis (CAPD) patients and identify influencing factors.

**Methods:**

Patients with end-stage renal disease were enrolled. Clinical data of iron metabolism and biochemical and dialysis parameters during the first peritoneal dialysis evaluation were collected. Serum ferritin (SF) and transferrin saturation (TSAT) levels were evaluated, and independent influencing factors were identified by correlation and regression analyses.

**Results:**

Of 1,128 adult CAPD patients, 41.2% had iron deficiency (ID), 15.7% had absolute iron deficiency, and 8.2% had functional iron deficiency. The average SF level was (276.8 ± 277.9) μg/L, and iron saturation was (29.8 ± 12.7)%. Additionally, 50.2 and 69.3% of patients reached targets in SF level and iron saturation recommended by the Chinese Society of Nephrology. SF level and TSAT were not correlated with estimated glomerular filtration rate, whereas negatively correlated with platelet count and inflammatory factors. Low platelet count, presence of diabetes mellitus and high interleukin 6 levels were independent factors of lower TSAT.

**Conclusions:**

ID is common in patients with CAPD. Women and those with thrombocytopenia, diabetes, and inflammation are at higher risk for iron storage or reduced iron utilization. In the initial CAPD stage, a reasonable iron supplement strategy may be established for CAPD patients with high-risk factors.

## 1 Introduction

Iron deficiency (ID) constantly occurs in dialysis patients. For these patients, iron intake is inadequate due to loss of appetite caused by uremia, dietary protein restriction, and drug side effects. These symptoms may be exacerbated by the presence of iron absorption and mobilization disorders [1]. The incidence of absolute iron deficiency (AID) and functional iron deficiency (FID) anemia is high in dialysis patients. Up to date, the management of anemia and iron status disturbances and outcomes in predialysis chronic kidney disease (CKD) and hemodialysis (HD) patients have been intensively studied. However, few studies analyzing the characteristics of anemia and iron metabolism in peritoneal dialysis (PD) patients have been conducted. Recent large-scale international study has demonstrated that more than half of PD patients have various degrees of anemia, and, regardless of iron supplementation, a certain percentage of them has disequilibrium of iron status [2]. Abnormal iron status is associated with an increased risk of all-cause and cardiovascular mortality. Furthermore, it is a factor in terms of both risk and prognosis for PD-related infectious complications [3,4]. In this study, clinical data of PD patients admitted to our hospital from 2011 to 2023 were retrospectively analyzed, and the indicators of iron metabolism and influencing factors in the early stages of ID were identified, aiming to provide a reference for developing anemia treatment strategies for patients with PD.

## 2 Subjects and methods

### 2.1 Inclusion and exclusion criteria

Patients who underwent PD catheterization for the first time in the Department of Nephrology, West China Hospital of Sichuan University, from January 2011 to June 2023 were recruited in this study. Inclusion criteria were as follows: (1) age >18 years and (2) the first follow-up assessment was performed 1–3 months after the start of PD. Exclusion criteria were as follows: (1) incomplete iron metabolism index data; (2) patients were undergoing HD simultaneously; and (3) patients receiving intravenous iron infusion or blood transfusion within 1 month.

### 2.2 Data collection

Clinical data were collected from the PD database for the initial evaluation of patients at 1–3 months after the initiation of their first PD. This initial evaluation included basic information, medications (oral iron, the use of erythropoiesis-stimulating agents [ESAs] and hypoxic inducible factor prolyl hydroxylase inhibitor – Roxadustat), iron metabolism index (serum ferritin [SF], serum iron, transferrin saturation [TSAT], and transferrin), hemoglobin, serum albumin, inflammation index (high-sensitivity C-reactive protein [hs-CRP], interleukin 6 [IL-6]), blood fat, parathyroid hormone (PTH), and electrolytes (blood calcium and phosphorus). Moreover, 24 h of PD fluid, 24 h of urine creatinine and urea concentration, 4 h of PD fluid creatinine concentration, and 24 h of urine volume and PD fluid in and out were collected to calculate dialyzing-related indexes. Body surface area (BSA) was calculated using the Gehan formula [5]. The assessment of PD included an estimate of glomerular filtration rate (eGFR) using an arithmetic mean of creatinine clearance and urea clearance. The weekly total urea clearance index (KT/V) and standardized protein nitrogen decomposition rate (nPCR) were calculated according to the adequacy guidelines recommended by the International Peritoneal Dialysis Association. The transport characteristics comprised 4-h PD and plasma creatinine ratio (D/P4h), calculated using Adequest 2.0, run by Baxter PD.

### 2.3 Definition

According to the “Chinese expert consensus on diagnosis and treatment for renal anemia (2018 revision)”, ID or relative ID was defined as SF < 100 μg/L or TSAT ≤ 20% [3]. AID was defined as SF < 100 μg/L and TSAT < 20% [4]. FID was defined as ferritin > 100 μg/L and TSAT < 20% [5,6]. SF was defined as 100 μg/L ≤ SF < 500 μg/L and TSAT attainment was defined as 20% ≤ TSAT < 50%, respectively.

### 2.4 Statistical analysis

All analyses were conducted with IBM SPSS Statistics version 20 (IBM Corp., NY, USA). At baseline, categorical data were compared using Chi-square test, and continuous variables were analyzed using an unpaired *t*-test or nonparametric Mann–Whitney *U* test. Data are presented as mean ± standard deviation and as median and range, where appropriate. Person correlation and Spearman correlation analyses were used for measurement data of normal distribution and non-normal distribution, respectively. Kendall correlation analysis was adopted for classification variables. Factors associated with iron metabolism (SF and TSAT) were screened by correlation analysis (inclusion criteria were <0.05). The screened correlation factors were taken as independent variables, and the independent influencing factors of SF and TSAT were analyzed by multivariate linear regression. *P* < 0.05 was considered statistically significant.


**Ethical approval:** This study has been approved by the Ethics Committee of West China Hospital, Sichuan University (Grant No. 891, 2019).
**Informed consent:** Informed written consent was obtained from the patients or their representatives.

## 3 Results

### 3.1 Baseline characteristics

A total of 1,187 eligible continuous ambulatory PD patients were eligible for the inclusion and exclusion criteria. Among them, 59 patients were excluded, including 50 (4.2%) with incomplete data, 5 (0.5%) with concurrent HD, and 4 (0.3%) with a history of intravenous iron infusion or/or blood transfusion within the last month. Finally, 1,128 eligible patients were enrolled. The mean age was (45.9 ± 14.0) years (18–91 years). A total of 739 cases (65.5%) were male, 881 patients (78.1%) received oral iron, 745 patients (66.0%) received erythropoietin, and 176 (15.6%) patients received Roxadustat (those initially received Roxadustat from January 2020 in our hospital). A total of 651 cases (57.7%) had hemoglobin levels of lower than 100 g/L. Clinical data of all patients are shown in Table 1. More women, lower BSA, and higher IL-6 levels were observed in the ID group than in the non-ID group.

**Table 1:** Comparison of baseline characteristics between the ID and non-ID groups

Index	Total	ID group, *N* = 456	Non-ID group, *N* = 672	*P*
Male (%)	739 (65.5%)	48.0%	68.75%	<0.001
Age (years)	45.66 (21.78)	45.22 (18.72)	44.81 (21.74)	0.39
BSA (m^2^)	1.66 ± 0.17	1.63 ± 0.17	1.67 ± 0.17	0.005
DM (%)	180 (16.0%)	17.30%	14.10%	0.149
Albumin (g/L)	35.42 ± 4.39	35.02 ± 4.92	34.88 ± 5.02	0.116
Prealbumin (g/L)	348.59 ± 85.09	342.75 ± 82.05	353.01 ± 87.06	0.115
Urea (mmol/L)	19.77 (7.453)	19.7 (7.34)	18.95 (7.50)	0.338
Creatinine (μmol/L)	694.45 (308.25)	716 (253.7)	731.35 (313)	0.3.3
Uric acid (μmol/L)	416.05 ± 85.50	418.01 ± 82.87	415.83 ± 89.34	0.659
Triglyceride (mmol/L)	1.33 (0.76)	1.32 (0.75)	1.36 (0.86)	0.139
Cholesterol (mmol/L)	4.45 (1.09)	4.56 (1.31)	4.43 (1.21)	0.213
Ca (mmol/L)	2.13 ± 0.22	2.13 ± 0.20	2.14 ± 0.23	0.741
P (mmol/L)	1.38 (0.49)	1.42 (0.51)	1.36 (0.54)	0.103
PTH (pmol/L)	19.32 (20.15)	18.49 (21.32)	18.82 (20.15)	0.089
Hemoglobin (g/L)	95.45 ± 18.65	95.07 ± 18.75	95.49 ± 18.09	0.776
Platelet count (×10^9^/L)	163.00 (75.57)	168.00 (74)	147.00 (73.75)	0.634
WBC count (×10^9^/L)	5.32 (1.83)	5.225 (1.93)	5.24 (2.24)	0.991
IL-6 (pg/mL)	6.06 (6.59)	5.93 (5.54)	5.28 (5.51)	<0.001
SF (μg/L)	195.00 (321.63)	51.24 (60.52.)	306.45 (329.69)	<0.001
Transferrin (g/L)	1.86 (0.58)	2.14 (0.52)	1.78 (0.44)	<0.001
Serum iron (μmol/L)	10.8 (6.32)	8.56 (4.92)	12.67 (5.62)	<0.001
TSAT (%)	27.12 (16.48)	18.2 (9.36)	33.56 (15.47)	<0.001
hsCRP (mg/L)	1.87 (4.89)	1.63 (3.71)	1.84 (3.68)	0.613
eGFR (mL/min)	4.23 (3.52)	3.68 (3.12)	3.78 (4.05)	0.315
nPCR	0.90 (0.25)	0.91 (0.28)	0.90 (0.27)	0.491
Weekly KT/V	2.04 (0.72)	2.04 (0.60)	2.02 (0.74)	0.365
D/P4h	0.67 ± 0.12	0.67 ± 0.12	0.68 ± 0.12	0.142
ESAs administration (%)	745 (66.0%)	63.8%	68.2%	0.257
Oral iron (%)	881 (78.1%)	76.2%	77.2%	0.918
Roxadustat (%)	176 (15.6%)	16.7%	15.9%	0.923

Note: BSA, body surface area; DM, diabetes mellitus; PTH, parathyroid hormone; WBC, white blood cell; eGFR, estimated glomerular filtration rate; IL-6, interleukin 6; SF, serum ferritin; nPCR, standardized protein nitrogen decomposition rate.

### 3.2 Iron metabolism and ID

**Figure 1:**
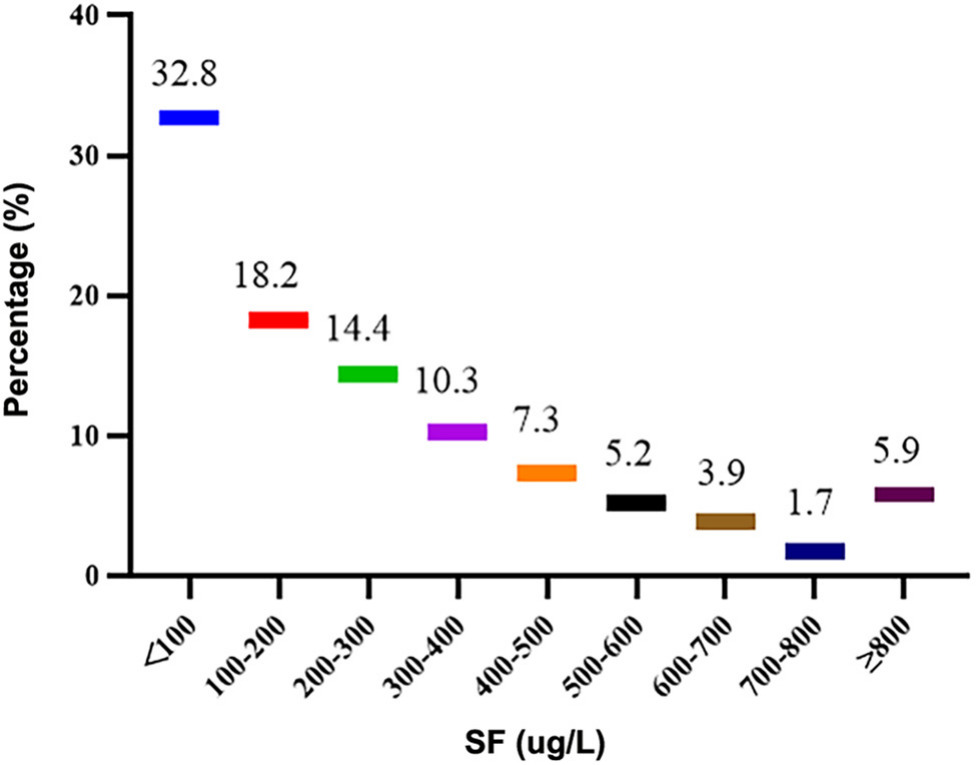
SF (μg/L) distribution in initial PD patients. 32.8% (370) patients had SF < 100 μg/L, 32.6% (368) patients had SF in 100–300 μg/L, and 5.9% (67) patients had SF ≥ 800 μg/L.

**Figure 2:**
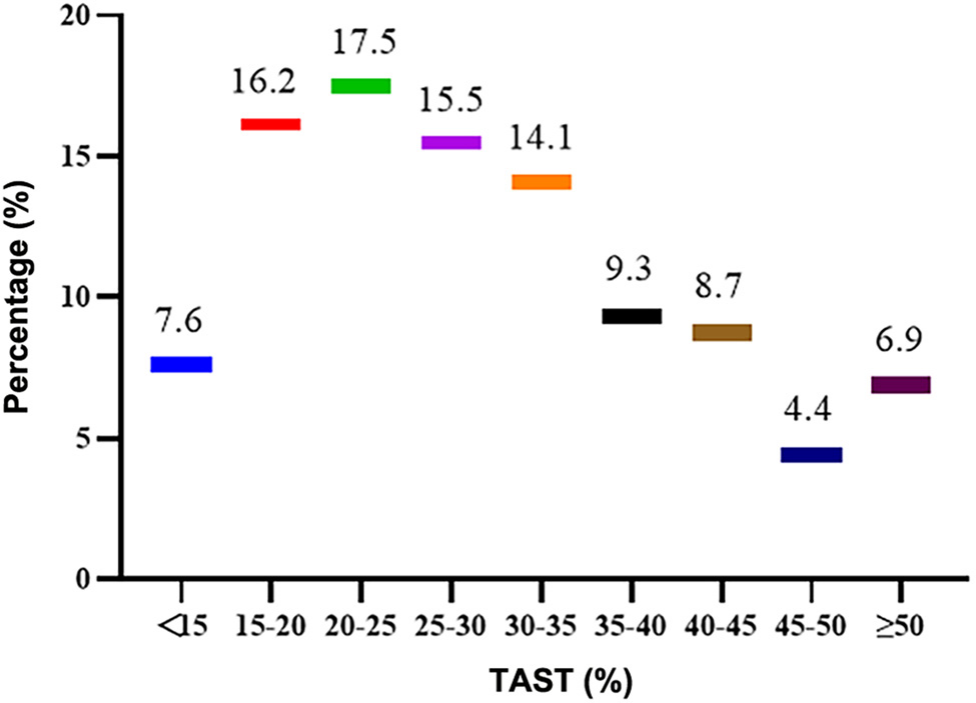
Ferritin saturation (%) distribution in initial PD patients. The TSAT achievement rate was 69.3% (782 cases), TSAT < 20% was in 23.8% (268 cases), and TSAT ≥ 50% was in 6.9% (137 cases).

The average SF was 276.8 ± 277.9 μg/L, with a median of 193.58 μg/L (interquartile spacing 319.68 μg/L), as shown in Figure 1. 32.8% (*n* = 370), 32.6% (*n* = 368), and 5.9% (*n* = 67) patients had SF < 100 μg/L, 100–300 μg/L, and ≥ 800 μg/L, respectively. The SF target rate was 50.2% (*n* = 566). The mean TSAT was (29.8 ± 12.7)%, with a median of 26.96% (interquartile spacing 16.12%) (Figure 2). The TSAT attainment rate was 69.3% (*n* = 782). TSAT < 20% in 23.8% (*n* = 268). 40.3% (*n* = 455) met the criteria of both serum SF and TSAT. 41.2% (*n* = 465) were diagnosed with relative ID, 15.7% (*n* = 177) had AID, and 8.1% (92 cases) had FID. Of patients with AID, 83.1% (*n* = 147) had taken oral iron. Among t881 patients treated with oral iron, 16.2% (*n* = 143) still had AID, and 39.6% (*n* = 349) had relative ID. Among 176 patients treated with Roxadustat, 48.9% (*n* = 86) had relative ID. Among them, 80.1% (*n* = 69) had SF < 100 μg/L.

### 3.3 Correlation analysis of iron metabolism index

Correlation analysis showed that SF was correlated with male (*r =* 0.208, *P* < 0.001) and was positively correlated with BSA, pre-albumin, and serum creatinine. Serum albumin, hemoglobin, total cholesterol, platelet count, hs-CRP, total KT/V, use of EPO, and Roxadustat were negatively correlated with SF. TSAT was associated with age, sex, diabetes mellitus, total cholesterol, platelet count, and IL-6 (Table 2).

**Table 2:** Correlation analysis of SF and ferritin saturation

	SF		Ferritin saturation	
	*r*	*P*	*r*	*P*
Gender	0.206	0.000	0.094	0.004
Age	−0.021	0.573	−0.133	0.001
BSA	0.1161	0.000	0.034	0.389
DM	0.014	0.612	−0.075	0.021
Albumin	−0.082	0.039	−0.014	0.721
Prealbumin	0.089	0.013	0.076	0.058
Urea	0.002	0.968	−0.026	0.521
Creatinine	0.123	0.002	0.069	0.082
Triglyceride	0.057	0.143	−0.012	0.768
Cholesterol	−0.086	0.024	−0.107	0.007
Calcium	−0.071	0.073	0.001	0.982
Phosphorus	−0.006	0.835	−0.04	0.32
PTH	0.032	0.544	0.004	0.925
Hemoglobin	−0.083	0.035	0.061	0.126
Platelet count	−0.173	0.000	−0.219	0.000
WBC count	0.013	0.761	−0.074	0.062
IL-6	0.056	0.168	−0.162	0.000
hs-CRP	0.161	0.000	−0.069	0.097
eGFR	−0.027	0.48	0.013	0.739
nPCR	−0.0622	0.128	0.025	0.533
Total KT/V	−0.115	0.003	−0.011	0.791
D/P4h	0.046	0.256	−0.004	0.911
ESAs	−0.067	0.035	−0.058	0.148
Oral iron	−0.056	0.147	0.012	0.768
Roxadustat	−0.024	0.027	0.035	0.576

### 3.4 Analysis of influencing factors of SF

In the multivariate linear regression analysis, SF was taken as the dependent variable. Gender, serum albumin, pre-albumin, serum creatinine, total cholesterol, hemoglobin, platelet count, hs-CRP, BSA, total KT/V, ESAs, and Roxadustat input were taken as the independent variables. Multivariate linear regression analysis demonstrated that gender, serum albumin, pre-albumin, platelet count, and hs-CRP were independently associated with SF (Table 3).

**Table 3:** Multivariate analysis of SF in initial PD patients

	B	SE	Beta	*t*	*P*	95% CI
Constant	525.735	185.497		2.834	0.005	161.379–890.09
Male	66.955	33.25	0.113	2.014	0.045	1.646–132.264
Cholesterol	−1.295	11.404	−0.005	−0.114	0.910	−23.695 to 21.105
Platelet count	−0.782	0.214	−0.16	−3.651	0.000	−1.203 to −0.361
Creatinine	0.072	0.053	0.068	1.361	0.174	−0.032 to 0.176
Albumin	−11.898	2.819	−0.208	−4.221	0.000	−17.434 to −6.361
Hemoglobin	−0.274	0.74	−0.018	−0.37	0.711	−1.728 to 1.18
Prealbumin	0.744	0.17	0.228	4.383	0.000	0.411 to 1.077
hs-CRP	4.673	1.021	0.199	4.578	0.000	2.668 to 6.677
KT/V	47.999	24.542	0.096	1.956	0.051	−0.208 to 96.205
ESAs	−41.859	23.821	−0.071	−1.757	0.079	−88.648 to 4.931
BSA	−69.508	89.305	−0.042	−0.778	0.437	−244.923 to 105.907
Roxadustat	−34.767	9.098	0.016	−1.531	0.528	−76.765–

### 3.5 Analysis of factors affecting TSAT

In multivariate linear regression analysis, TSAT was utilized as the dependent variable and associated factors (age, gender, diabetes mellitus, total cholesterol, platelet count, and IL-6) showed that platelet count, diabetes mellitus, and IL-6 were the independent factors of TSAT (Table 4).

**Table 4:** Multivariate analysis of TSAT in initial PD patients

	B	SE	Beta	*t*	*P*	95% CI
Constant	37.653	3.195		11.784	0	31.377–43.93
Male	2.153	1.129	0.083	1.907	0.057	−0.065 to 4.372
Diabetes	−3.338	1.521	−0.099	−2.194	0.029	−6.326 to −0.35
CHOL	−0.478	0.506	−0.041	−0.945	0.345	−1.472 to 0.516
Age	−0.027	0.041	−0.031	−0.669	0.504	−0.108 to 0.053
Platelet count	−0.028	0.009	−0.132	−3.099	0.002	−0.045 to −0.010
IL-6	−0.059	0.024	−0.104	−2.479	0.013	−0.105 to −0.012

## 4 Discussion

PD is one of the alternative therapies for patients with end-stage renal failure. Despite the widespread use of ESAs, the incidence of anemia in PD patients is still high. In recent years, Roxadustat is also extensively chosen. Roxadustat is a novel agent with a distinct mechanism of action compared to ESAs and a potentially different combination of effects on iron parameters [6,7,8]. In 2012, the investigation results of 9 large dialysis centers in China showed that the incidence of anemia in 613 PD patients was 53.5% [9]. ID is the most common cause of low ESA response [10]. Few investigations have been carried out regarding the iron metabolisms of PD patients in China. In the present study, the SF and TSAT attainment rates of initial PD patients were 50.2 and 69.3%, respectively. 40.3% of patients reached both SF and TSAT attainment rates. The mean SF values were decreased in patients with initial PD who received Roxadustat in this study, which is consistent with the results of previous studies [6,7,8]. Of the patients treated with Roxadustat who had relative ID, 80.1% had SF < 100 μg/L. Significantly greater reductions in SF occurred following Roxadustat use in patients with non-dialysis-dependent (NDD) CKD compared to placebo use and in two of three studies in patients with dialysis-dependent (DD) CKD relative to ESAs use [11,12]. In a pooled analysis of three studies in NDD CKD patients and three studies in DD CKD patients from the ALPINE program, in patients with NDD CKD, SF was initially decreased through week 8 following Roxadustat initiation and gradually increased, but it remained below the baseline level. In contrast, patients with DD CKD experienced an initial decrease in SF at week 8 and subsequent gradual decreases through week 52 that were similar to, though greater than, decreases seen with ESAs.

In addition, 78.1% of the patients received oral iron supplements. Intravenous iron infusions were rare and only applied in less than 1% of patients (only three cases of intravenous iron infusion were screened in this study). In contrast to PD, intravenous iron is more widely used in HD patients, with 41–82% of HD patients using intravenous iron [2]. The reasons for PD patients rarely requiring iron infusions include PD patients not needing to go to the hospital frequently, having no ready vascular channel, the need to be hospitalized for intravenous iron transfusion unlike oral iron, etc., which reduces the compliance of PD patients with intravenous iron supplementation and limits the application and utility of intravenous iron. ID was still common in patients with initial PD, with 41.2% having relative ID and 15.7% having AID. International guidelines recommend oral iron supplementation for PD patients, the same as for CKD patients without dialysis. However, in this study, 16.2% of patients with oral iron supplementation were found to be AID. FID refers to normal or increased storage of iron, but an ID in cells is associated with inflammation. In this study, only 8.1% of PD patients had FID, lower than those in patients with HD and CKD. Previous studies reported that the prevalence of FID (defined as TSAT < 20% and SF > 200 μg/L) was 23% in HD patients [13], which may be related to the slighter inflammatory state of PD than HD [14]. The prevalence of FID (defined as TSAT < 20% and SF 100–300 μg/L) was 12% at stages 2 and 3 of CKD [15]. These differences may be caused by the study population, FID definition and iron treatment strategies. In terms of SF distribution, SF < 100 μg/L, <300 μg/L, and ≥800 μg/L of PD patients in this study were 32.8, 65.4, and 5.9%, respectively, similar to Japan (44, 83, and 3%) but vary significantly from the United States [2]. In the United States, only 5% of PD patients had SF less than 100 μg/L, while 39% had SF > 800 μg/L [2]. The high SF levels observed in the United States are probably caused by different management strategies. Although the recommended timing or low target line of iron supplementation was almost the same worldwide, the recommended upper limit of iron supplementation was different when SF < 100 μg/L. In China, the recommended upper limit of SF is 500 μg/L, while in Japan, the upper limit is more strict to avoid reaching >300 μg/L. In the United Kingdom, >800 μg/L is avoided, and in the United States, there is no upper limit. The low SF in this study is consistent with China’s guidelines for renal anemia. TSAT < 20% was observed in 23.8% of patients in this study, similar to Australia/New Zealand, Canada, and the United Kingdom (24–27%), but higher than in the United States (13%) and Japan (11%) [2]. No consensus has been reached on the reasonable target range of SF and TSAT.

Correlation and multivariate regression analyses indicated that women were more likely to have low SF, which was consistent with reports of low iron reserves in women in the general population and may be related to menstruation and vaginal bleeding [16]. SF was negatively correlated with serum albumin, whereas positively correlated with pre-albumin level. The correlation between serum albumin and pre-albumin, both nutritional indexes, and SF was contradictory. Among nutritional biomarkers, serum albumin is by far the most widely studied serum protein in maintenance dialysis patients [17]. However, serum albumin and pre-albumin are also affected by inflammation. The utility of using low serum albumin and low pre-albumin as a rationale for diagnosing malnutrition has been questioned in recent years. Reduced albumin and pre-albumin levels should not be considered a sign of malnutrition in the presence of an acute response (e.g., elevated CRP) when they serve as negative markers of acute response. Albumin has a longer half-life of about 21 days, while pre-albumin has a shorter half-life of about 1.9 days, which is similar to C-reactive protein, which has a half-life of 46.4 h. The shorter half-life of pre-albumin is likely a better indicator of recent nutritional status and inflammatory properties, while serum albumin’s longer half-life may better reflect nutritional reserves. Therefore, we believe that nutrient reserves are negatively correlated with SF, which may be due to protein consumption during the synthesis of SF or hemoglobin. Alternatively, it could also be related to disease states. Inflammatory cytokines including IL-6 and hs-CRP were independently associated with TSAT and SF, respectively, which is consistent with previous findings in the literature [18]. Increased hepatic iron regulation during inflammation inhibits intestinal iron absorption and release from macrophages, reducing iron reserves and iron utilization [19]. Iron metabolism was negatively correlated with platelet count. Earlier literature also features similar reports. In anemia patients with chronic disease, lower serum iron levels are associated with higher platelet counts [20]. Animal studies have shown increased platelet reactivity in ID anemia [21]. Intravenous iron supplementation has also been shown to reduce platelet count in CKD. Animal studies have reported that iron can inhibit the maturation of megakaryocytes. When iron is deficient, inhibition is weakened, the differentiation of megakaryocytes is accelerated, and megakaryocytes have higher ploidy, which leads to increased platelet production [21]. ID can cause secondary thrombocytopenia and platelet activation and even lead to thrombosis in patients with chronic diseases [14,22]. Iron supplementation and anticoagulant therapy can prevent the occurrence or recurrence of thrombosis in patients with ID [22]. However, ferritin levels were not associated with platelet count in healthy individuals [23]. Whether thrombocytopenia can be a predictor of ID in PD patients needs further study.

## 5 Study limitations

Several limitations have to be acknowledged in this study. First, all subjects in this study are initial PD patients. Although oral iron is generally absorbed quickly, relevant iron indexes may not reach a stable level immediately. Second, this study is a single-center retrospective study, with multiple biases such as selectivity. Third, differences in dietary habits may also lead to differences in iron intake. Hence, the results only reflect the PD population in the western region of China.

## 6 Conclusion

Taken together, the prevalence of ID remains high in patients with initial PD. Oral iron supplementation alone may not treat ID in some patients with initial PD. Roxadustat use is correlated with a greater reduction in SF levels compared with ESAs and an increase in serum iron levels compared to a decrease with ESAs. A decline in TSAT in patients treated with Roxadustat is relatively small. These changes can reflect the increases in both serum iron and total iron-binding capacity. Iron metabolism is affected by gender and inflammation levels, and it is independently associated with platelet count. Increased platelet count may be a predictor of ID. SF levels should be fully assessed before using Roxadustat, and iron reserves should be appropriately increased. In our future research, we plan to design a prospective randomized controlled multi-center study and stratify patient age, aiming to further validate the conclusion that thrombocytopenia can be a predictor of ID in PD patients.

## Abbreviations

CAPD: continuous ambulatory peritoneal dialysis
ESRD: end-stage renal disease
ID: iron deficiency
AID: absolute iron deficiency
FID: functional iron deficiency
HD: hemodialysis
PD: peritoneal dialysis
ESAs: erythropoiesis-stimulating agents
HIF-PHI: hypoxic inducible factor prolyl hydroxylase inhibitor
SF: serum ferritin
TSAT: transferrin saturation
hs-CRP: high-sensitivity c-reactive protein
IL-6: interleukin 6
PTH: parathyroid hormone
BSA: body surface area
eGFR: estimate of glomerular filtration rate
NDD: non-dialysis dependent
CKD: chronic kidney disease
DD: dialysis dependent
